# Ischemic Preconditioning Mediates Neuroprotection against Ischemia in Mouse Hippocampal CA1 Neurons by Inducing Autophagy

**DOI:** 10.1371/journal.pone.0137146

**Published:** 2015-09-01

**Authors:** Chunlin Gao, Ying Cai, Xuebin Zhang, Huiling Huang, Jin Wang, Yajing Wang, Xiaoguang Tong, Jinhuan Wang, Jialing Wu

**Affiliations:** 1 Department of Neurology, Tianjin Huanhu Hospital, Tianjin Key Laboratory of Cerebrovascular and Neurodegenerative Diseases, Tianjin, China; 2 Department of Neuroscience, Tianjin Huanhu Hospital, Tianjin Key Laboratory of Cerebrovascular and Neurodegenerative Diseases, Tianjin Neurosurgery Institute, Tianjin, China; 3 Department of Pathology, Tianjin Huanhu Hospital, Tianjin Key Laboratory of Cerebrovascular and Neurodegenerative Diseases, Tianjin, China; 4 Department of Neurosurgery, Tianjin Huanhu Hospital, Tianjin Key Laboratory of Cerebrovascular and Neurodegenerative Diseases, Tianjin Neurosurgery Institute, Tianjin, China; University of Kentucky, UNITED STATES

## Abstract

The hippocampal CA1 region is sensitive to hypoxic and ischemic injury but can be protected by ischemic preconditioning (IPC). However, the mechanism through which IPC protects hippocampal CA1 neurons is still under investigation. Additionally, the role of autophagy in determining the fate of hippocampal neurons is unclear. Here, we examined whether IPC induced autophagy to alleviate hippocampal CA1 neuronal death in vitro and in vivo with oxygen glucose deprivation (OGD) and bilateral carotid artery occlusion (BCCAO) models. Survival of hippocampal neurons increased from 51.5% ± 6.3% in the non-IPC group (55 min of OGD) to 77.3% ± 7.9% in the IPC group (15 min of OGD, followed by 55 min of OGD 24 h later). The number of hippocampal CA1 layer neurons increased from 182 ± 26 cells/mm^2^ in the non-IPC group (20 min of BCCAO) to 278 ± 55 cells/mm^2^ in the IPC group (1 min × 3 BCCAO, followed by 20 min of BCCAO 24 h later). Akt phosphorylation and microtubule-associated protein light chain 3 (LC3)-II/LC3-I expression were increased in the preconditioning group. Moreover, the protective effects of IPC were abolished only by inhibiting the activity of autophagy, but not by blocking the activation of Akt in vitro. Using in vivo experiments, we found that LC3 expression was upregulated, accompanied by an increase in neuronal survival in hippocampal CA1 neurons in the preconditioning group. The neuroprotective effects of IPC on hippocampal CA1 neurons were completely inhibited by treatment with 3-MA. In contrast, hippocampal CA3 neurons did not show changes in autophagic activity or beneficial effects of IPC. These data suggested that IPC may attenuate ischemic injury in hippocampal CA1 neurons through induction of Akt-independent autophagy.

## Introduction

Ischemic preconditioning (IPC) is a brief sublethal ischemic event occurring in neurons regulated by endogenous cellular protective mechanisms, generating subsequent prolonged resistance to ischemic injury. Multiple studies have confirmed the presence of brain tissue IPC and elucidated the molecular mechanisms involved in IPC, including the release of adenosine [1], increase of nitric oxide and bradykinin [2], activation of the K^+^-ATP channel [3], downregulation of inflammatory gene expression [4], improvement of endothelial function [5], and other factors [6–8].

Autophagy is an important process in eukaryotic cells. Under conditions of hypoxia or lack of nutrients, autophagy enables cells to effectively use biodegradable materials, such as aggregates from degenerated or damaged proteins and redundant or damaged organelles (mitochondria), thereby providing energy to support protein synthesis [9]. Autophagy has been shown to reduce ischemic damage in the brain, liver, heart, kidney, lung, and other tissues [10–12]. Recent studies have shown that autophagy is involved in IPC-induced protection during cerebral ischemia [13,14].

The hippocampus is a critical brain region involved in learning and memory. IN the hippocampus, the CA1 layer is the most sensitive to hypoxic and ischemic injury [15]. IPC has been shown to alleviate damage in hippocampal CA1 layer neurons in the bilateral carotid artery occlusion (BCCAO) mouse model [16]. Moreover, IPC can induce increased autophagy in hippocampal CA1 neurons [17]. However, to the best of our knowledge, the mechanism through which IPC protects hippocampal CA1 neurons is poorly understood.

In this study, we examined these mechanisms in oxygen glucose deprivation (OGD) and BCCAO mouse models. Our results indicated that the activation of Akt-independent autophagy induced by IPC conferred protection on hippocampal CA1 neurons during cerebral ischemia.

## Materials and Methods

### Materials

Pregnant C57BL/6J mice (17–18 days of gestation) and adult mice (6–8 weeks old, 25–30 g) were purchased from the Experimental Animal Center of Peking Union Medical College. Experiments were approved by the Institutional Animal Care and Use Committee of Tianjin Huanhu Hospital. l-Glutamine, 3-methyladenine (3-MA), poly-d-lysine, and the 3-(4,5-dimethylthiazol-2-yl)-2,5-diphenyltetrazolium bromide (MTT) assay kit were purchased from Sigma Company (St. Louis, MO, USA). Rabbit anti-mouse LC3, Akt, and phospho-Akt antibodies were purchased from Cell Signaling Technology (Danvers, MA, USA). Enhanced chemiluminescence (ECL) reagents were purchased from Millipore Company (USA). Fetal bovine serum (FBS), neurobasal medium, B-27 serum-free supplement, Dulbecco’s modified Eagle’s medium (DMEM)/F-12 medium, trypsin, rapamycin, and wortmannin were purchased from Life Technologies (Carlsbad, CA, USA). The Akt inhibitor GDC-0068 was purchased from Selleckchem. Glucose-free DMEM was purchased from Gibco Company (USA).

### Hippocampal Neuronal Cultures, Exposure to OGD Conditions, and Determination of Cell Survival

Hippocampal neurons were isolated from pregnant C57BL/6J mice (at 17–18 days of gestation) as described elsewhere [16,18]. Briefly, the fetal hippocampal tissue was separated and transferred into Hanks’ balanced salt solution containing 100 units/mL penicillin, 100 μg/mL streptomycin, and 10 mm HEPES. After careful dissection, the hippocampal tissue pieces were incubated in 0.125% trypsin solution at 37°C for 10 min and flowed through a 200-mesh sieve. Tissues were then centrifuged at 1000 rpm for 10 min, and the precipitate was resuspended in B27-supplemented neurobasal medium and plated onto 0.04 mg/mL poly-d-lysine-coated wells. Previous studies have shown that 55 min of OGD induces cell death in approximately 50% of hippocampal neurons [16,19]. Thus, cultured hippocampal neurons were exposed to OGD conditions (< 0.1% oxygen, 5% CO_2_, and 94.9% N_2_ in glucose-free DMEM) for different times (5, 10, 15, 30, or 55 min) and tested 24 h later by MTT assay to screen out the lethal OGD time of 55 min. The effects of IPC on neuronal death were then studied. Hippocampal neurons were exposed to OGD for different times (5, 10, 15, or 30 min), followed by another 55 min of OGD 24 h later. Cell survival was then determined 24 h later using MTT assays. Results are expressed as the percentage of cell survival relative to that in control cells maintained under normoxic conditions. A subgroup of cells was treated with IPC and 100 nM 3-MA, IPC and 10 nM GDC-0068, or 10 nM rapamycin under lethal OGD conditions. To evaluate the biological effects of these treatments on neuronal survival, another subgroup of cells was treated with 100 nM 3-MA, 10 nM rapamycin, or 10 nM GDC-0068 under normoxic conditions. Each experiment was repeated 6–8 times.

### Animal Model of Cerebral Ischemia and IPC

To induce IPC in brain ischemic conditions, a BCCAO mouse model was used [16,20]. Briefly, C57BL/6 mice were anesthetized with 4% isoflurane and intubated with a small-animal respirator. A midline incision was made, and bilateral common carotid arteries were carefully isolated and occluded by artery clips. After 1, 2, 10, or 20 min, the clips were removed to restore cerebral blood flow, and the incision was closed. In groups subjected to artery occlusion for 1 or 2 min, the blood flow occlusion-restore procedure was repeated three times at intervals of 5 min. Body temperature was maintained at 37°C throughout the procedure and recovery using a heating pad. After 24 h, the mice were sacrificed, and the brain tissues were collected. To determine the protective effects of IPC against ischemic damage in hippocampal neurons, all mice in the IPC and control groups received another 20 min of BCCAO at 24 h after the first surgery (n = 6 per group). To investigate changes in autophagic activities and the associated mechanisms involved in mediating the protective effects of IPC on the brain, another subgroup of mice subjected to IPC was randomly selected to receive intraventricular injection with 3-MA (25 nM, 5 μL), 2 μL wortmannin (0.1 mM), or an equivalent amount saline (n = 8 per group), at bregma 0 mm, mediolateral 2 mm, and dorsoventral 3 mm, 2 h before the first surgery [21–23]. After the second BCCAO procedure, all mice were sacrificed either 6 h later for immunohistochemical staining or 24 h later for Nissl staining.

### Western Blotting

After extraction of total protein, the protein concentration was determined using Bradford assays, and 10 μg of total protein was loaded for sodium dodecyl sulfate polyacrylamide gel electrophoresis (SDS-PAGE). Immunoblotting was performed with antibodies directed against LC3, phospho-Akt, total Akt, and β-actin.

### Immunohistochemical Staining

Mice were deeply anesthetized and transcardially perfused with 4% paraformaldehyde at 6 h after lethal BCCAO. The brains were incubated sequentially in 10%, 20%, and 30% sucrose solutions overnight, embedded in paraffin, and sectioned to 5 μm thickness through the dorsal and ventral hippocampus using a vibratome. After deparaffinization/rehydration, antigen was retrieved using 10 mM sodium citrate buffer. Next, sections were blocked with 10% goat serum for 1 h at room temperature, followed by incubation overnight at 4°C with primary antibodies against LC3 (1:1000 dilution). Subsequently, antibodies were detected with the EnVision Detection System (DK-2600; DAKO, Glostrup, Denmark). Three areas of interest (AOIs) in the hippocampal CA1 and CA3 layers were chosen. Each observation was repeated five times. Images were captured using a Nikon ECLIPSE 80i microscope with Nikon Plan Fluor lenses (20× and 40× magnification; Nikon, Tokyo, Japan).

### Nissl Staining Analysis

For Nissl staining, 5-μm-thick paraffin-embedded sections were incubated with 1% cresylviolet at 50°C for 20 min. After rinsing with double distilled water, sections were dehydrated and mounted with Permount. The hippocampal CA1 and CA3 layers from each animal were analyzed. Six visual fields (0.6 mm^2^) of CA1 and CA3 layers were photographed in each section. The number of stained cells in each field was counted at higher magnification (4×, 20×, and 40×). The data were represented as the number of cells per mm measured using the scale-bars under a high-power field.

### Statistical Analysis

Data are presented as the mean ± standard error of the mean (SEM). Unless stated otherwise, we carried out statistical analysis with SPSS 13.0 statistical software using two-tailed Student’s t-tests if two groups were compared or one-way analysis of variance (ANOVA) with Bonferroni’s multiple comparisons post-hoc test for comparisons of more than two groups. Differences were with *p* values of less than 0.05 were considered significant.

## Results

### Protective Effects of IPC on Hippocampal Neuronal Death during Ischemic Insult

**Fig 1:**
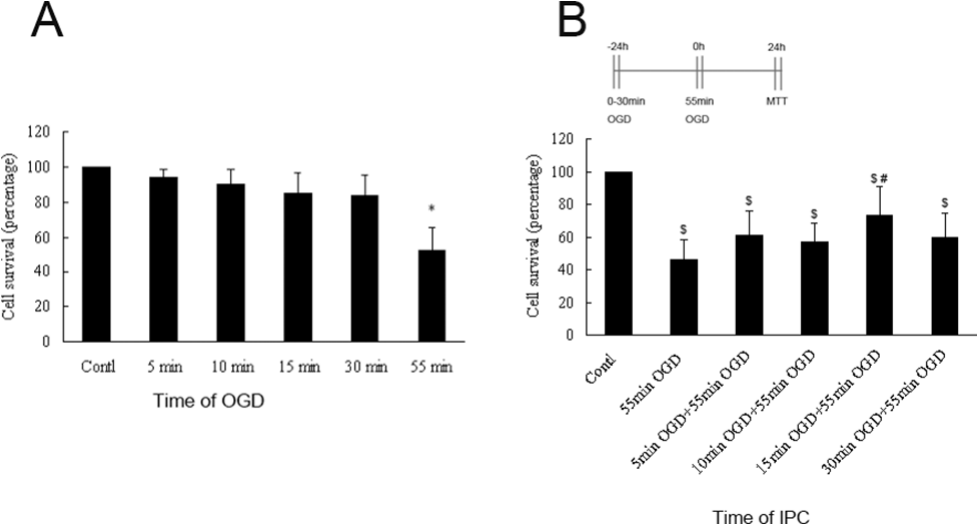
Determination of the lethal OGD time and suitable IPC intervention in hippocampal neurons. (A) Time-dependent effects of hypoxia on neuronal death. Hippocampal neurons were exposed to OGD conditions for 0, 5, 10, 15, 30, or 55 min, followed by quantification of cell survival with MTT assays 24 h later. (B) Different OGD times conferred protection against the deleterious effects of 55 min of OGD. Hippocampal neurons were exposed to sublethal (0–30 min) OGD followed by 55 min of lethal OGD 24 h later. Values are given as percentages compared with cell survival in neurons maintained under normoxic conditions. Error bars denote SDs. *P < 0.01 compared with cells maintained under OGD for 0–30 min. ^**$**^ *P* < 0.01 compared with cells maintained under normoxic conditions. ^#^ *P* < 0.05 compared with cells maintained under 55 min of OGD.

Hippocampal neurons were exposed to 0–55 min of OGD, followed by determination of cell survival by MTT assays 24 h later. Our results showed that exposure to 5, 10, 15, or 30 min of OGD stimulus and reperfusion for 24 h did not cause significant neuronal death. In contrast, exposure to 55 min of OGD decreased neuronal survival to 52% ± 12.9%, significantly lower relative to survival percentages after 0–30 min of OGD (*p* < 0.01; Fig 1A); therefore, the lethal OGD time was set as 55 min. To confirm whether different OGD times altered neuronal vulnerability to lethal OGD, we exposed hippocampal neurons to sublethal OGD for different times (5, 10, 15, or 30 min), followed by lethal OGD (55 min of OGD) 24 h later. We found that 15 min of OGD conferred protection against the deleterious effects of lethal OGD by increasing neuronal survival to 73% ± 18.2% (*p* < 0.05, Fig 1B). Therefore, we chose stimulation with 15 min OGD as the suitable IPC intervention in the subsequent experiments.

### IPC Conferred Protection to Hippocampal CA1 Layer Neurons but Not CA3 Layer Neurons

**Fig 2:**
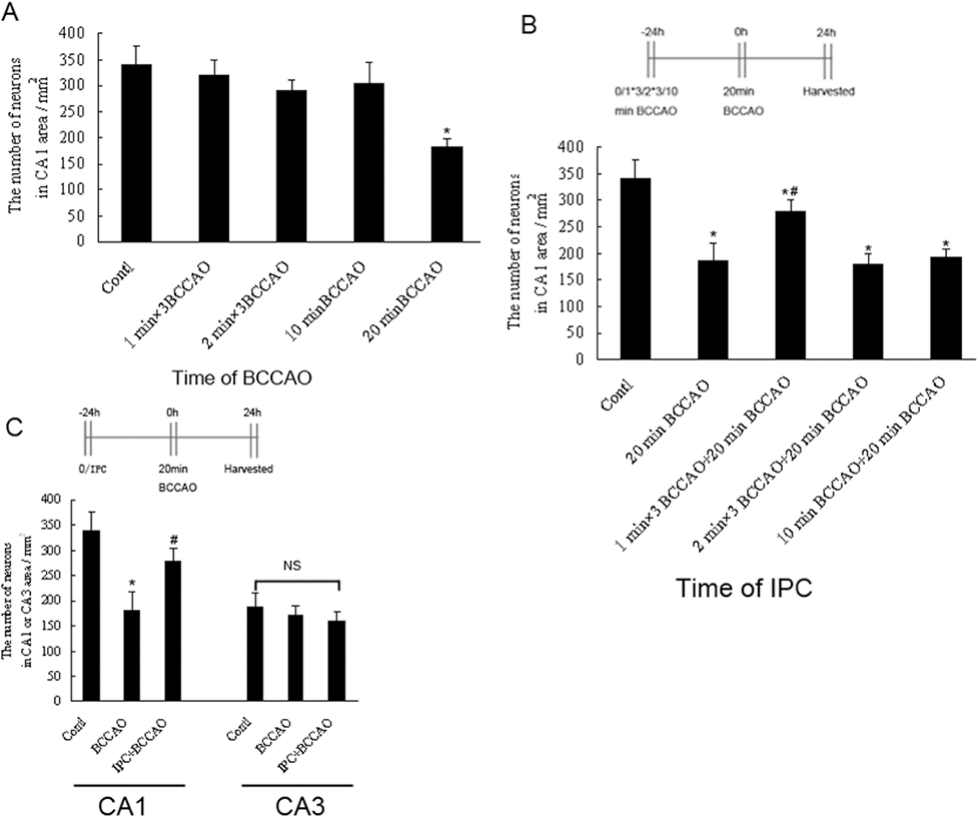
Determination of the lethal BCCAO time and the effects of IPC against lethal BCCAO in hippocampal CA1 neurons. (A) The effects of different types of BCCAO on survival of neurons in the hippocampal CA1 layer. Mice were exposed to several patterns of BCCAO followed by quantification of cell survival with Nissl staining 24 h later. (B) Different patterns of BCCAO protected CA1 neurons against 20 min of BCCAO. Mice were exposed to several patterns of sublethal BCCAO followed by 20 min lethal BCCAO 24 h later, and quantification of cell survival was carried out using Nissl staining 24 h later. (C) Comparison of the vulnerability of the CA1 and CA3 regions of the hippocampus to preconditioning. Values are given as the mean number of neurons. Error bars denote SDs. *P < 0.01 compared with the control group. ^#^ *P* < 0.05 compared with other experimental groups.

Because the CA1 region of the hippocampus was more vulnerable to ischemia than the CA3 region, we decided to assess the role of IPC in neurons in the hippocampal CA1 and CA3 layers during cerebral ischemia in vivo. We first determined the time of lethal BCCAO and suitable IPC. We performed Nissl staining 24 h after three episodes of BCCAO (1 or 2 min in duration, separated by 5-min intervals of normal perfusion) or 10 or 20 min of BCCAO. Nissl staining showed that there were no significant differences in the number of neurons in the hippocampal CA1 layer in control, 1 min × 3 BCCAO, 2 min × 3 BCCAO, and 10 min BCCAO groups (340 ± 58, 320 ± 40, 289 ± 32, and 305 ± 62 cells/mm^2^, respectively; *p* > 0.05). In contrast, the number of CA1 layer neurons was significantly reduced to 182 ± 26 cells/mm^2^ after exposure to 20 min of BCCAO (*p* < 0.01 compared with other subgroups). Therefore, we set 20 min as the lethal BCCAO time (Fig 2A). In cells exposed to 1 min × 3 BCCAO + 20 min BCCAO, the number of hippocampal CA1 neurons was 278 ± 55 cells/mm^2^, representing a significant increase (*p* < 0.01) compared with those in the 20 min BCCAO group. Significant differences were also observed compared with the 2 min × 3 BCCAO + 20 min BCCAO and 10 min BCCAO + 20 min BCCAO groups (180 ± 20 and 193 ± 15 cells/mm^2^, respectively; *p* < 0.05). Therefore, we set 1 min × 3 BCCAO as the suitable IPC time (Fig 2B).

Nissl staining showed that there were no significant differences in the numbers of neurons in the hippocampal CA3 layer in the control, 20 min of BCCAO, and preconditioning groups (188 ± 34, 170 ± 28, and 160 ± 26 cells/mm^2^, respectively; *p* > 0.05). In contrast with the CA1 layer, 20 min of BCCAO failed to induce extensive neuronal death, and IPC did not have a protective role in the CA3 layer (Fig 2C).

### The Neuroprotective Role of Autophagy Induced by IPC in Hippocampal Neuronal Cells

**Fig 3:**
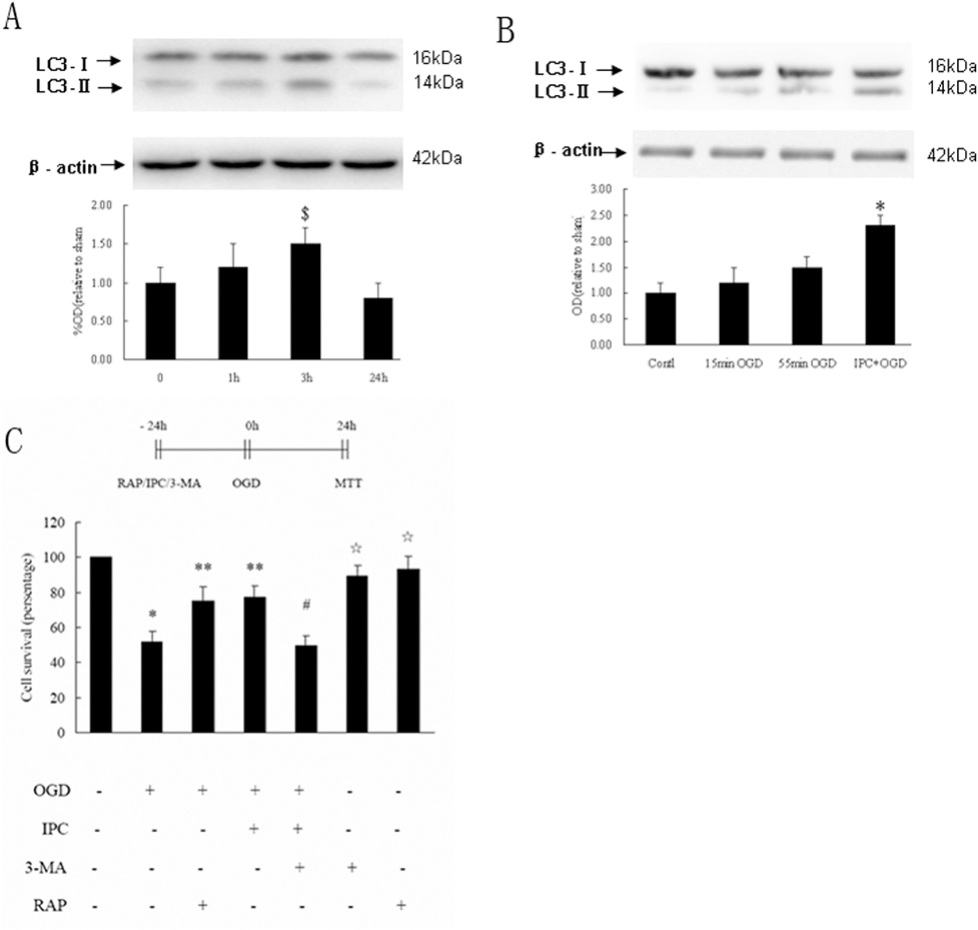
Autophagic activation induced by IPC protected hippocampal neurons. (A) Representative image of western blot analysis of LC3 and β-actin expression in lysates from hippocampal neurons at different times (0, 1, 3, or 24 h) after exposure to lethal OGD in the preconditioning group. (B) Western blot analysis of LC3 and β-actin expression in hippocampal neurons 3 h after exposure to normoxic conditions, 55 min of OGD alone, or 15 min of OGD followed by 55 min of OGD 24 h later. (C) Mean survival in hippocampal neurons exposed to 55 min of OGD; pretreated with IPC alone, 10 nM rapamycin alone, or IPC plus 100 nM 3-MA 24 h before, or pretreated with 100 nM 3-MA alone or 10 nM rapamycin alone under normoxic conditions. Error bars denote SDs. ^$^ *P* < 0.05 compared with the other groups. *P < 0.05 compared with cells under normoxic conditions. **P < 0.05 compared with neurons maintained under 55 min of OGD. ^#^ *P* < 0.05 compared with cells in the preconditioning group. ^☆^ *P* > 0.05 compared with cells maintained under normoxic conditions.

Many studies have indicated that autophagy protects neurons from cerebral ischemia [10–12]. Therefore, we evaluated whether autophagy mediated the protective effects of IPC. LC3-II is a well-established marker of autophagosomes in mammalian cells. Therefore, we examined changes in LC3-II/LC3-I at the molecular level. First, we performed western blot analysis for LC3-II/LC3-I in the preconditioning group. Cell lysates from hippocampal neurons were collected at different times (0, 1, 3, or 24 h) after exposure to lethal OGD. We found that LC3-II/LC3-I expression began to rise at 1 h, reach a peak at 3 h, and returned to normal levels 24 h later (Fig 3A). Therefore, we chose 3 h after exposure to lethal OGD as the time point for evaluation of autophagy. Hippocampal neurons were collected 3 h after exposure to normoxic conditions (control), 15 min sublethal OGD followed 24 h later by exposure to lethal OGD conditions (i.e., the preconditioning group), or lethal OGD. IPC induced a rapid increase in LC3-II/LC3-I expression (*p* < 0.05), whereas no significant differences in LC3-II/LC3-I expression were observed in groups exposed to 15 min of sublethal OGD or lethal OGD compared with the control (Fig 3B).

Mammalian target of rapamycin (mTOR) is a vital negative regulator of autophagy. Therefore, we next performed MTT assays to investigate the effects of changes in mTOR-dependent autophagic activity on hippocampal neuronal survival by promoting autophagy using the mTOR inhibitor rapamycin or inhibiting autophagy by treatment with 3-MA. The survival of hippocampal neurons after 55 min of OGD increased from 51.5% ± 6.3% in nonpreconditioned cells to 77.3% ± 7.9% in preconditioned neurons or to 75.1% ± 6.6% in neurons pretreated with 10 nM rapamycin (*p* < 0.05). Interestingly, pretreatment with a combination of IPC and 100 nM 3-MA completely abolished the protective effects of IPC (49.3% ± 6.2%). To rule out the adverse effects of 3-MA and rapamycin, we examined neuronal survival following incubation with 100 nM 3-MA alone or 10 nM rapamycin alone under normoxic conditions. The neuronal survival rates were 89.5% ± 5.3% and 93.4% ± 6.2% in cells treated with 3-MA alone or rapamycin alone, respectively (*p* > 0.05; Fig 3C).

### IPC-Induced Autophagy Protected Neurons in the Hippocampal CA1 Layer but Not in the CA3 Layer

**Fig 4:**
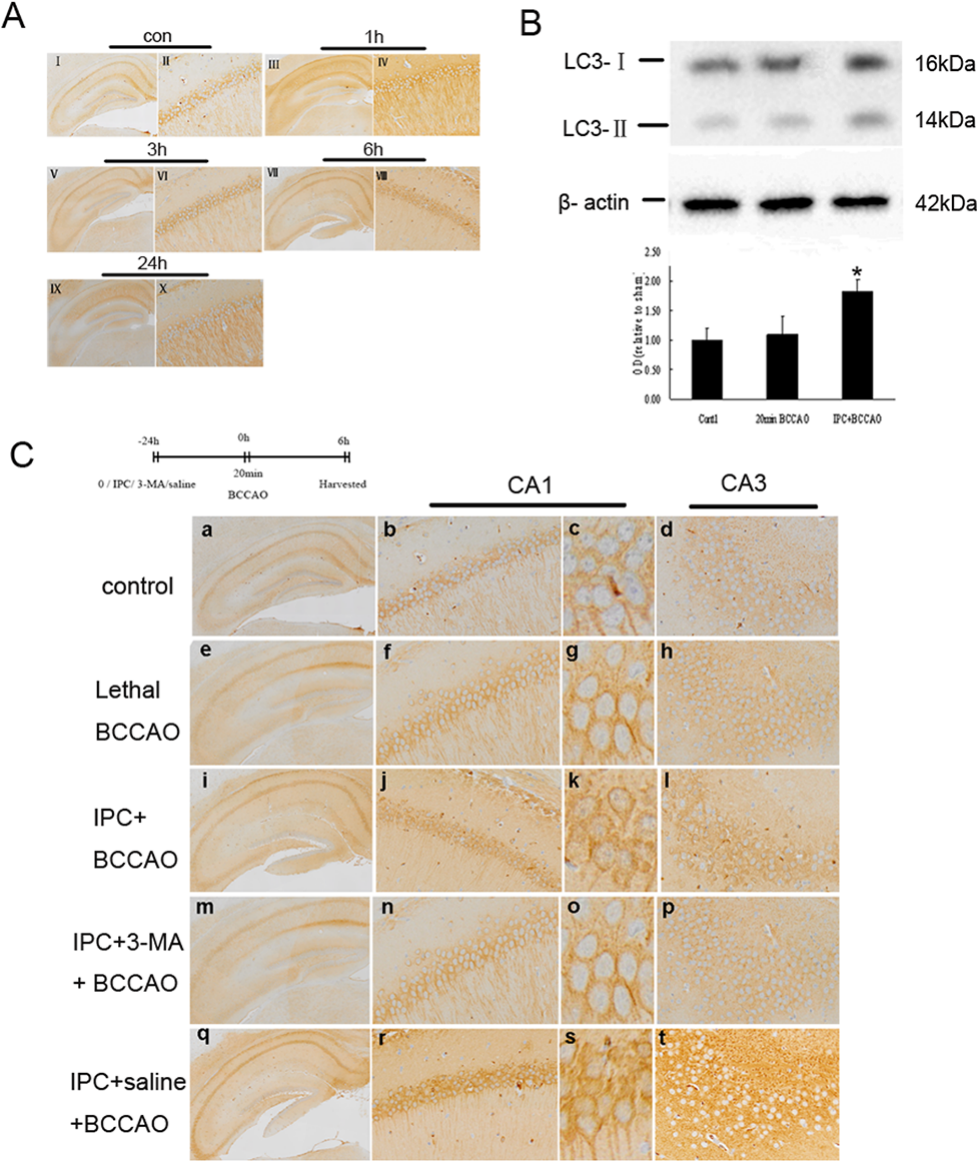
Protective effects of IPC-induced autophagy against lethal BCCAO in the hippocampal CA1 layer. (A) Mice were exposed to control (I, II) or 1 min × 3 sublethal BCCAO followed by 20 min lethal BCCAO 24 h later. The distribution of LC3 was then quantified in hippocampal CA1 neurons by immunohistochemistry at 1 (III, IV), 3 (V, VI), 6 (VII,VIII), and 24 h (IX,X). (B) Mice were exposed to 20 min lethal BCCAO alone, or 1 min × 3 sublethal BCCAO followed by 20 min lethal BCCAO 24 h later. Western blot analysis of LC3 and β-actin expression in mice hippocampus 6 h after lethal BCCAO. (C)Mice were exposed to 1 min × 3 sublethal BCCAO, IPC plus 3-MA, or IPC plus saline, followed by 20 min lethal BCCAO 24 h later. The distribution of LC3 was then quantified in hippocampal area (a, e, i, m, q), and CA1 neurons (b, c, f, g, j, k, n, o, r, s) and CA3 neurons (d, h, l, p, t) 6 h later. Immunohistochemistry showed a strong increase in LC3 expression at 6 h after the lethal BCCAO in the preconditioning group (j, k) and after pretreatment with IPC and saline (r, s) compared with that of the control (b, c) and lethal BCCAO alone (f, g). However, the increase in LC3 expression was inhibited following pretreatment with a combination of IPC and 3-MA (n, o). High-magnification views revealed the presence of numerous LC3-positive dots in hippocampal CA1 neurons only in the preconditioning subgroup (k) and following pretreatment with IPC and saline (s), but not in the control (c) or following 20 min of lethal BCCAO (g) or pretreatment with IPC and 3-MA (o). No changes in LC3 expression were detected in the CA3 layer (d, h, l, p, t). Original magnification, 2× (I, III, V, VII, IX,a, e, i, m, and q), and 10× (II, IV, VI, VIII, X, b, d, f, h, j, l, n, p, r and t) and 100× (c, g, k, o, and s). Error bars denote SDs. *P < 0.01 compared with the control group.

Because our in vitro results indicated that IPC activated autophagy to protect hippocampal neurons and conferred protection to the hippocampal CA1 layer but not the CA3 layer, we speculated that the neuroprotective role of IPC in hippocampal CA1 neurons was dependent on autophagy. In the preconditioning group, LC3 labeling increased as early as 3 h after lethal BCCAO (Fig 4A, VI), reached a maximum at 6 h (Fig 4A, VIII), and returned to normal levels at 24 h (Fig 4A, X). Therefore, we choose 6 h after the experiment as the time point for evaluation of autophagy.

Because the main index for autophagy is the ratio of LC3-II/LC3-I, it is unclear whether the increased expression of total LC3 is also an indicator of autophagic activation. Therefore, we next evaluated autophagic activity in our in vivo model. We found that LC3-II/LC3-I expression was also increased in the preconditioning group compared with that in the control group and the lethal BCCAO alone group (Fig 4B).

LC3 labeling was diffuse, faint, and homogenously distributed in hippocampal CA1 and CA3 neurons in the control group (Fig 4C, b and d). Mice were exposed to 1 min × 3 sublethal BCCAO alone, with a combination of IPC and intraventricular injection of 3-MA, or with IPC and intraventricular injection of saline followed by 20 min lethal BCCAO 24 h later. Autophagy was then evaluated 6 h later, and neuronal survival was evaluated 24 h later in the hippocampal CA1 and CA3 layers. Preconditioning (Fig 4C, j) and pretreatment with a combination of IPC and saline (Fig 4C, r) induced a rapid increase in LC3 expression. In contrast, no significant changes in LC3 expression were observed after 20 min lethal BCCAO (Fig 4C, f) or pretreatment with IPC and 3-MA (Fig 4C, n) compared with that in the control (Fig 4C, b). High-magnification views indicated the presence of numerous LC3-positive dots in hippocampal CA1 neurons only in mice subjected to preconditioning (Fig 4C, k) or pretreatment with IPC and saline (Fig 4C, s), but not in control mice (Fig 4C, c) or mice subjected to 20 min of lethal BCCAO (Fig 4C, g) or pretreatment with a combination of IPC and 3-MA (Fig 4C, o). No changes in LC3 expression were found in neurons in the CA3 layer (Fig 4C, d, h, l, p, and t).

**Fig 5:**
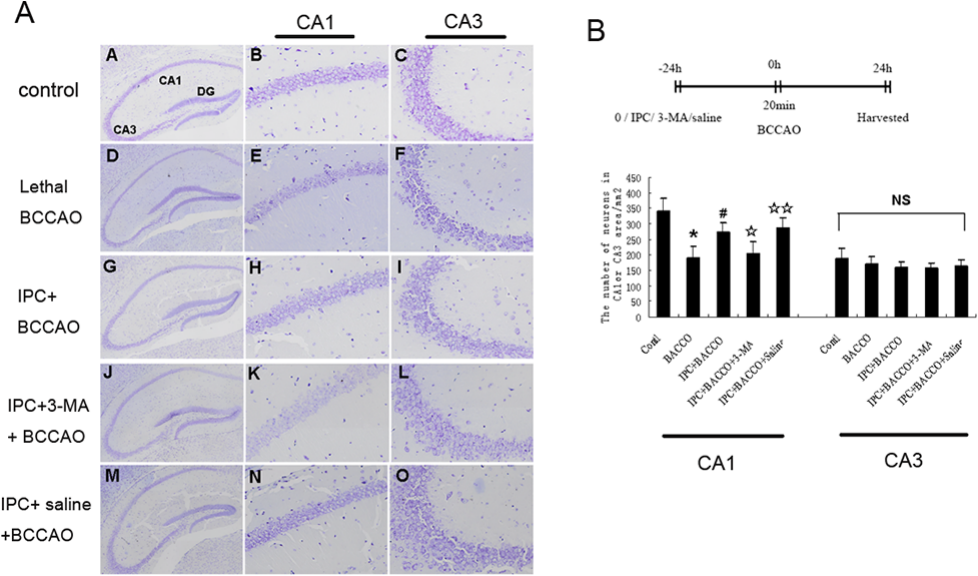
The neuroprotective effects of IPC-induced autophagy in the hippocampal CA1 layer. Representative images of Nissl staining of the hippocampus. Mice were exposed to 1 min × 3 sublethal BCCAO, a combination of IPC and intraventricular injection of 3-MA, or IPC and intraventricular injection of saline followed by 20 min lethal BCCAO 24 h later. Cell survival was then quantified by Nissl staining in hippocampal CA1 (A, B, D, E, G, H, J, K, M, and N) and CA3 (C, F, I, L, and O) layers 24 h later. Values are given as the mean number of neurons. Error bars denote SDs. *P < 0.01 compared with the control group. ^#^ *P* < 0.05 compared with the nonpreconditioning group. ^**☆**^ *P* < 0.01 compared with the preconditioning group. ^**☆☆**^ *P* > 0.05 compared with the preconditioning group. Original magnification, 2× (A, D, G, J, and M) and 10× (B, C, E, F, H, I, K, L, N, and O).

Nissl staining showed that the number of hippocampal CA1 neurons increased from 190 ± 37 cells/mm^2^ in the nonpreconditioning group to 272 ± 32 cells/mm^2^ in the preconditioning group. However, the numbers of living cells following treatment with a combination of IPC and 3-MA or IPC and saline before lethal BCCAO were 205 ± 36 and 285 ± 35 cells/mm^2^, respectively, in neurons in the hippocampal CA1 layer, indicating that inhibition of autophagy blocked the beneficial effects of IPC. Importantly, we did not observe any protective effects of IPC in the hippocampal CA3 layer after ischemic insult in our in vivo BCCAO model (*p* > 0.05; Fig 5).

### The Role of Akt Phosphorylation on the Activation of Autophagy Induced by IPC

**Fig 6:**
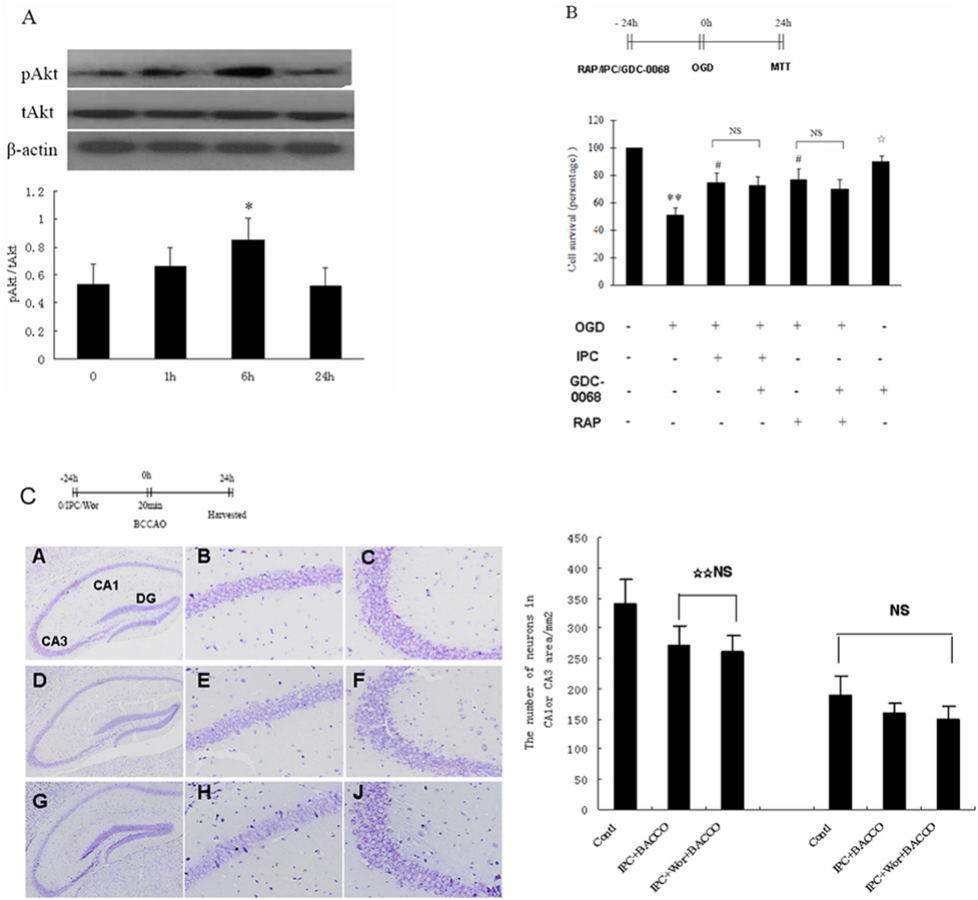
IPC-induces autophagy conferred neuroprotection independent of Akt phosphorylation. (A) Representative image of western blot analysis for Akt phosphorylated at serine 473 (pAkt), total Akt (tAkt), and β-actin in lysates from hippocampal neurons 6 h after exposure to normoxic conditions, 15 min of OGD alone, 55 min of OGD alone, or preconditioning. (B) Mean survival in hippocampal neurons exposed to 55 min of OGD; pretreated with IPC alone, 10 nM rapamycin alone, a combination of IPC and 10 nM GDC-0068, or a combination of 10 nM rapamycin and 10 nM GDC-0068 24 h before 55 min of OGD; or pretreated with 10 nM GDC-0068 alone under normoxic conditions. (C) Representative images of Nissl staining of the hippocampus. Mice were exposed to 1 min × 3 sublethal BCCAO or with a combination of IPC and intraventricular injection of wortmannin followed by 20 min of lethal BCCAO 24 h later. Cell survival was quantified by Nissl staining of the hippocampal CA1 and CA3 layers 24 h later. Values are given as the mean number of neurons. Error bars denote SDs. *P < 0.05 compared with other groups. **P < 0.05 compared with cells under normoxic conditions. ^#^ *P* < 0.05 compared with neurons maintained under 55 min of OGD. ^**☆**^ *P* > 0.05 compared with the control. ^**☆☆**^ *P* < 0.05 compared with the control.

Although IPC can protect neurons through induction of Akt phosphorylation [7], Akt phosphorylation has been shown to be a negative regulator of autophagy. Therefore, we examined whether IPC induced the activation of autophagy in an Akt-dependent manner in our IPC model. We performed western blot analysis to measure Akt phosphorylation at serine 473 (phospho-Akt) in the preconditioning group, in which cell lysates prepared from hippocampal neurons were collected at different times (0, 1, 6, or 24 h) after exposure to lethal OGD. We found that Akt phosphorylation began to rise at 1 h, peaked at 6 h (*p* < 0.05), and returned to baseline levels at 24 h (Fig 6A). Next, we used GDC-0068, a highly selective ATP-competitive Akt inhibitor, to evaluate the role of Akt phosphorylation on autophagy-mediated hippocampal neuronal survival in the OGD model in vitro. The survival of hippocampal neurons after 55 min of OGD increased from 50.7% ± 5.8% in nonpreconditioned cells to 74.6% ± 6.9% in preconditioned neurons and to 76.8% ± 8.2% in neurons pretreated with 10 nM rapamycin. Moreover, pretreatment with a combination of IPC and 10 nM GDC-0068 or 10 nM rapamycin and 10 nM GDC-0068 did not block the protective effects of IPC or autophagy (70.1% ± 6.8% and 72.2% ± 6.9%, respectively). Neuronal survival following incubation with 10 nM GDC-0068 under normoxic conditions remained unchanged compared with the control (92.2% ± 5.8%; Fig 6B). In the preconditioning group, pretreatment with intraventricular injection of wortmannin did not alter LC3 expression or improve neuronal survival in the hippocampal CA1 layer. Moreover, in hippocampal CA1 and CA3 neurons treated with a combination of IPC and intraventricular injection of wortmannin before lethal BCCAO, cell numbers (260 ± 27 and 148 ± 22 cells/ mm^2^, respectively) did not differ significantly compared with those of the preconditioning group (*p* > 0.05; Fig 6C).

## Discussion

In this study, we aimed to examine whether IPC induces autophagy to prevent the death of hippocampal CA1 neurons. Our data demonstrated that IPC attenuated ischemic injury in hippocampal CA1 neurons by inducing Akt-independent autophagy. Thus, these data provide important insights into the mechanisms of IPC.

The hippocampal CA1 region is known to be sensitive to hypoxic and ischemic injury [15,24]. Interestingly, we found the hippocampal CA3 neurons were less vulnerable to ischemia than CA1 neuronal cells and were not protected by IPC in our in vivo model. Lehotský et al. found that hippocampal CA1 neurons can be protected by IPC [8,25]. Additionally, some studies have shown that increased autophagic activity is an important mechanism protecting against ischemic injury in hippocampal CA3 neurons and that CA1 neurons, which lack this mechanism, have poor tolerance to ischemia [17,26]. While we demonstrated that IPC induced protection against hypoxic-ischemic injury in hippocampal CA1 neurons, the mechanism through which this process occurred was not clarified. We speculate that there may be some causal link among IPC, autophagic activation, and protection of hippocampal CA1 neurons. In agreement with other studies [13,14], our results indicated that pretreatment with sublethal IPC before lethal OGD and BCCAO could upregulate LC3-II/LC3-I in vitro and LC3 in the hippocampal CA1 layer in vivo.

Autophagy is a “double-edged sword”; moderate autophagy can accelerate the degradation of intracellular proteins errors and promote cell survival, whereas excessive activation of autophagy can lead to cell lysis and autophagic cell death [10]. Autophagy can be either beneficial or harmful in brain ischemia [17,27–31]. Indeed, studies have found that LC3-II expression increases over time after hypoxic-ischemic injury in mice [18]. Moreover, application of 3-MA inhibits autophagy via the phosphatidylinositol 3-kinase (PI3K)/Akt pathway, thereby increasing ischemic brain injury, whereas rapamycin reduces ischemic brain damage. In contrast, Cui et al. found that 3-MA inhibits autophagy and apoptosis and significantly reduces infarct volume after focal cerebral ischemia in mice [30]. Hypoxic-ischemic injury has also been shown to induce the activation of autophagy in hippocampal neurons, subsequently triggering hippocampal neuronal death, and mice deficient in the autophagy gene *Atg7* exhibit significantly reduced neuronal death [31]. This paradox reflects the complexity of the relationship between autophagy and ischemic brain damage, both of which are affected by the degree of macrophage activation, the duration of ischemia, and differences in experimental models of ischemia [17]. Ginet et al found that in contrast to CA3 neurons, CA1 neurons exhibit only a minimal increase in autophagy but have strong apoptotic characteristics, suggesting that the enhancement of autophagy plats a neuroprotective role in delayed neuronal death after ischemic insults in the CA1 region [26]. However, no studies have shown whether activation of autophagy via IPC affects the survival of hippocampal CA1 neurons during ischemic injury. Consistent with other studies, our in vitro experiments demonstrated the neuroprotective role of autophagy in hippocampal neurons [16,26]. Importantly, treatment with the mTOR antagonist rapamycin, which has been shown to increase autophagy, had the same protective effect as IPC, and treatment with 3-MA, which has been shown to inhibit autophagy, completely abolished the neuroprotective effects of IPC. In this study, we used a BCCAO model to evaluate the CA1 layer specificity of the neuroprotective effects of IPC-induced autophagy. Our results showed that LC3-II/LC3-I expression was increased and that the number of hippocampal CA1 neurons also increased significantly in the preconditioning group compared with that in mice exposed to lethal BCCAO alone. Similar results were found in vitro; inhibition of autophagy with 3-MA completely abolished the neuroprotective effects of IPC. Interestingly, however, there were no changes in autophagic activity or neuronal survival in response to IPC in the hippocampal CA3 layer.

Some studies have shown that IPC-induced Akt phosphorylation can protect neurons [7,32]. Additionally, Akt phosphorylation also can block autophagy. In this study, we aimed to further examine the mechanisms through which IPC induces autophagy via Akt. Treatment with GDC-0068, a highly selective ATP-competitive Akt inhibitor [33], did not inhibit the protective effects of IPC in hippocampal neurons. Moreover, GDC-0068 did not block the neuroprotective effects of rapamycin. We hypothesize that induction of autophagy by IPC may occur independent of Akt, indicating that IPC-induced Akt phosphorylation and autophagic activation are relatively independent processes. This hypothesis is supported by previous studies showing that rapamycin provides neuroprotective effects against cerebral ischemia through the concomitant activation of autophagy and the PI3K/Akt-mTOR axis [34,35].

There are several limitations to our study. There are several limitations to our study. First, we did not clarify the connections among IPC-induced autophagy, Akt phosphorylation, and neuroprotection, and we may not have identified all of the neuroprotective effects of IPC. Second, for technical reasons, it is difficult to dissect the hippocampal CA1 layer from the hippocampus, and we could not evaluate the differences in neuronal death between the hippocampal CA1 and CA3 layers during ischemic injury in vitro. Further studies in mice with knockout of autophagy-related genes are needed to confirm the neuroprotective effects of IPC against ischemia in specific hippocampal areas.

In conclusion, we propose that IPC exerts neuroprotective effects in hippocampal CA1 neurons through the activation of an Akt-independent autophagic signaling pathway. Our findings have important clinical relevance, revealing new mechanisms of neuroprotection in the ischemic brain. Moreover, our results indicate that regulation of autophagic activity may be a potential therapeutic strategy for the management of patients with acute ischemic stroke.

## Supporting Information

S1 File NC3Rs ARRIVE Guidelines Checklist 2014. (PDF)
